# Comparison of renal histopathology and gene expression profiles between severe COVID-19 and bacterial sepsis in critically ill patients

**DOI:** 10.1186/s13054-021-03631-4

**Published:** 2021-06-10

**Authors:** Meint Volbeda, Daniela Jou-Valencia, Marius C. van den Heuvel, Marjolein Knoester, Peter J. Zwiers, Janesh Pillay, Stefan P. Berger, Peter H. J. van der Voort, Jan G. Zijlstra, Matijs van Meurs, Jill Moser

**Affiliations:** 1 Department of Critical Care, University of Groningen, University Medical Center Groningen, Hanzeplein 1, 9713 GZ Groningen, The Netherlands; 2 Department of Pathology and Medical Biology, Pathology Section, University of Groningen, University Medical Center Groningen, Groningen, The Netherlands; 3 Department of Clinical Microbiology and Infection Prevention, University of Groningen, University Medical Center Groningen, Groningen, the Netherlands; 4 Department of Pathology and Medical Biology, Medical Biology Section, Laboratory for Endothelial Biomedicine and Vascular Drug Targeting Research, University of Groningen, University Medical Center Groningen, Groningen, The Netherlands; 5 Department of Internal Medicine, University of Groningen, University Medical Center Groningen, Groningen, The Netherlands

**Keywords:** COVID-19, Acute kidney injury, Bacterial sepsis

## Abstract

**Background:**

The mechanisms driving acute kidney injury (AKI) in critically ill COVID-19 patients are unclear. We collected kidney biopsies from COVID-19 AKI patients within 30 min after death in order to examine the histopathology and perform mRNA expression analysis of genes associated with renal injury.

**Methods:**

This study involved histopathology and mRNA analyses of postmortem kidney biopsies collected from patients with COVID-19 (*n* = 6) and bacterial sepsis (*n* = 27). Normal control renal tissue was obtained from patients undergoing total nephrectomy (*n* = 12). The mean length of ICU admission-to-biopsy was 30 days for COVID-19 and 3–4 days for bacterial sepsis patients.

**Results:**

We did not detect SARS-CoV-2 RNA in kidney biopsies from COVID-19-AKI patients yet lung tissue from the same patients was PCR positive. Extensive acute tubular necrosis (ATN) and peritubular thrombi were distinct histopathology features of COVID-19-AKI compared to bacterial sepsis-AKI. ACE2 mRNA levels in both COVID-19 (fold change 0.42, *p* = 0.0002) and bacterial sepsis patients (fold change 0.24, *p* < 0.0001) were low compared to control. The mRNA levels of injury markers NGAL and KIM-1 were unaltered compared to control tissue but increased in sepsis-AKI patients. Markers for inflammation and endothelial activation were unaltered in COVID-19 suggesting a lack of renal inflammation. Renal mRNA levels of endothelial integrity markers CD31, PV-1 and VE-Cadherin did not differ from control individuals yet were increased in bacterial sepsis patients (CD31 fold change 2.3, *p* = 0.0006, PV-1 fold change 1.5, *p* = 0.008). Angiopoietin-1 mRNA levels were downregulated in renal tissue from both COVID-19 (fold change 0.27, *p* < 0.0001) and bacterial sepsis patients (fold change 0.67, *p* < 0.0001) compared to controls. Moreover, low Tie2 mRNA expression (fold change 0.33, *p* = 0.037) and a disturbed VEGFR2/VEGFR3 ratio (fold change 0.09, *p* < 0.0001) suggest decreased microvascular flow in COVID-19.

**Conclusions:**

In a small cohort of postmortem kidney biopsies from COVID-19 patients, we observed distinct histopathological and gene expression profiles between COVID-19-AKI and bacterial sepsis-AKI. COVID-19 was associated with more severe ATN and microvascular thrombosis coupled with decreased microvascular flow, yet minimal inflammation. Further studies are required to determine whether these observations are a result of true pathophysiological differences or related to the timing of biopsy after disease onset.

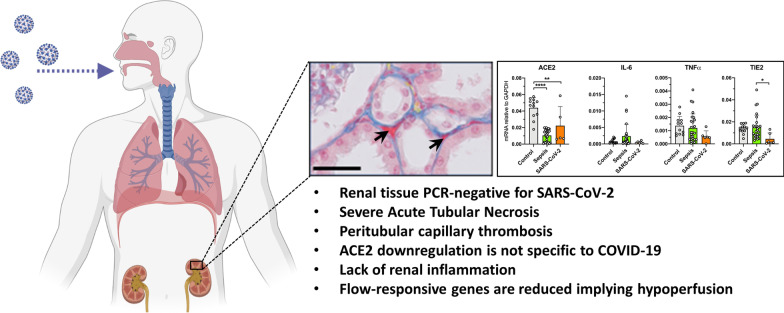

**Supplementary Information:**

The online version contains supplementary material available at 10.1186/s13054-021-03631-4.

## Introduction

The recent COVID-19 pandemic has resulted in major morbidity and mortality. The number of ICU-admitted COVID-19 patients developing AKI (COVID-AKI) varies per geographical area but has been reported to be as high as 20–76% [1–4] and is associated with poor prognosis [5]. The mortality of critically ill COVID-19 patients requiring renal replacement therapy (RRT) varies between 42 and 90% [4, 6, 7]. Although recent autopsy studies have provided information on the consequences of SARS-CoV-2 infection on kidney histopathology, the mechanisms driving renal failure remain poorly understood and treatment strategies aiming to limit or reverse renal failure are still lacking. Previous reports have suggested that the renal pathophysiology associated with COVID-19 is similar to that described for patients with bacterial sepsis [8, 9]. However, studies investigating the differences and similarities have not yet been reported.

The aim of this study was therefore to collect postmortem kidney biopsies from COVID-19 patients rapidly after death in order to examine the histopathology and to integrate molecular analyses in order to gain an in-depth understanding of the possible mechanisms of renal injury and failure associated specifically with severe SARS-CoV-2 infection. In light of the discussion regarding the similarities between COVID-19 and bacterial sepsis, we compared bacterial sepsis data from previously published studies by our group [10–12] with new data from COVID-19 patients. To our knowledge, this is the first study to report renal mRNA data which combined with histopathology has enabled us to identify at least some of the molecular mechanisms associated with COVID-AKI.

## Materials and methods

### Human postmortem biopsies

#### Control

Normal control renal biopsies were collected from a healthy part of the kidney cortex from patients that underwent complete nephrectomy for kidney cancer (*n* = 12). Renal tissue residing as far away from the tumor as possible was collected. A renal pathologist at our hospital (MvdH) assessed these “normal control” biopsies and considered them to be normal kidney tissue.

#### Bacterial sepsis

Postmortem kidney biopsies were collected from adult patients with bacterial sepsis (*n* = 27) as described in detail elsewhere [10]. Patients with bacterial sepsis who had pre-existing chronic kidney disease, active autoimmune disorders with renal involvement, or were treated with immune-suppressive medication were excluded from this study. All patients were classified as having septic shock according to the International Sepsis Definition [13]. The extent of renal failure was classified according to the, at that time, applicable RIFLE (Risk, Injury, Failure, Loss of kidney function, and End-stage kidney disease) criteria [14].

#### COVID-19

Postmortem kidney and lung biopsies were collected from adult patients with SARS-CoV-2 infection (*n* = 6). The diagnosis of COVID-19 was confirmed by RT-PCR of oropharyngeal and nasopharyngeal swabs. The presence of SARS-CoV-2 was also determined in the sputum of patients. The timeline of hospitalization and SARS-CoV-2 detection is detailed in Fig. 2a. The extent of renal failure was classified according to the latest KDIGO (Kidney Disease: Improving Global Outcomes) criteria [15].

Clinical characteristics are described in Table 1. The rapid collection of postmortem biopsies was considered a limited autopsy. Permission and written informed consent were asked for, and obtained, in the final family meeting. All biopsies were taken as quickly as possible after death at the bedside in our ICU and are therefore considered “warm”. The Medical Ethics Review Committee (METC) of the University Medical Center Groningen (UMCG) reviewed this study. The Central ethics Review Board non-WMO studies (CTc UMCG) has approved this study (METc 2011/372 and Research Register Number: 201900431).

**Table 1:** Patient characteristics

	Control (*n* = 12)	Sepsis (*n* = 27)	COVID-19 (*n* = 6)
Age	59 (20–79)	67 (40–85)	65 (49–78)
Male:female	5:7	17:10	4:2
SAPS II score	n/a	68.8 (35–99)	
BMI (kg/m^2^)		26.9 (20.8–49)	30.8 (26–37)
Normal weight (BMI 18.5–24.9)		9	0
Overweight (BMI 25–29.9)		14	3
Obese (BMI > 30)		4	3
Days in ICU	n/a	3.6 (1–12)	30 (21–53)
Comorbidities			
Hypertension	3	8	1
Diabetes Mellitus	1	3	2
COPD or asthma	4	7	0
Coronary disease	1	5	0
Neurological	1	3	0
Renal disease	0	0	0
Vascular surgery	0	2	0
Autoimmune disease	2	4	0
Neoplasms (extra renal)	4	4	0
RIFLE stage			
Risk	n/a	0	n/a
Injury	n/a	12	n/a
Failure	n/a	15	n/a
Loss of function	n/a	0	n/a
End-stage renal disease	n/a	0	n/a
KDIGO AKI score			
0	n/a	n/a	1
1	n/a	n/a	0
2	n/a	n/a	0
3	n/a	n/a	5
Need for RRT (Yes:no)	n/a	12:15	4:2
Mean biopsy time (mins)	n/a	33 (24–150)	21 (6–40)
Microorganisms			
Gram positive	n/a	12	3^#^
Gram negative	n/a	23	1*
Virus (norovirus & HIV)	n/a	2	0
Fungi/yeast	n/a	5	0
SARS-CoV-2	n/a	n/a	6

*ICU* intensive care unit, *SAPS II *Simplified Acute Physiology Score II scoring system, *BMI* body mass index, *RIFLE* (risk, injury, failure, loss of kidney function, and end-stage kidney disease) classification, *KDIGO AKI* Kidney Disease Improving Global Outcomes acute kidney injury classification, *RRT* renal replacement therapy, *COPD *chronic obstructive pulmonary disease, *HIV* human immunodeficiency virus, *SARS-CoV-2* severe acute respiratory syndrome coronavirus 2. *n/a *not applicable. Microorganisms were detected in either the sputum, blood, urine or abdominal fluid

*Multi-resistant pseudomonas in blood and sputum.

^#^CVL or ECLS cannula-related infection with a coagulase-negative staphylococci

### Tissue sampling and histopathology

Multiple kidney biopsy tissue specimens were harvested from patients with bacterial sepsis and COVID-19 under ultrasound guidance after introducing the biopsy device (Angiotech, 14 Ga × 20 cm, Tru Core II Biopsy Instrument, Gainesville, Fl) through a small skin incision. Lung biopsies were also collected from 4 of the COVID-19 patients. Biopsy tissues were either immediately snap-frozen or placed in RNA*later®* (ThermoFisher Scientific, Bleiswijk, The Netherlands) solution and subsequently stored at − 80 °C until RNA isolation and gene expression analysis. For histopathological analysis, additional renal biopsies were immediately fixed in 10% formalin fixative for 24–48 h and subsequently processed and imbedded in paraffin. Hematoxylin and eosin, periodic acid-Schiff, Jones methenamine silver, and Martius scarlet, blue stains were performed on paraffin sections obtained from all patients and controls. All sections were evaluated and scored by the same experienced renal pathologist (MvdH) following the routine pathology procedure and therefore could not be blinded. Scoring methods were performed as described previously [10] and are summarized in Additional file 1: Table S1.

### RNA Isolation

Total RNA was isolated from human kidney cryosections or homogenized renal and lung biopsies using either a RNeasy Mini plus Kit or miRNAeasy Mini kit (Qiagen, Venlo, The Netherlands) according to the manufacturer's instructions. RNA concentration and purity were measured using a NanoDrop® ND-1000 ultraviolet spectrophotometer (NanoDrop Technologies, Rockland, DE), while RNA integrity was determined by gel electrophoresis.

### SARS-CoV-2 detection by rRT-PCR

Five microliter (µl) of extract with known RNA content (ng/µl) was combined with 5 µl DNAse/RNAse free water (Sigma, The Netherlands). SARS-CoV-2 rRT-PCR was performed on the E-gen and N-gen as described [16], with minor modifications. The PCR reaction is a multiplex for SARS-CoV-2 and Phocine Distemper Virus (PDV) and is performed in a total reaction volume of 25 µl using 10 µl input and 15 µl PCR mix, containing 1xTaqMan® Fast Virus 1-Step Master Mix (Applied Biosystems, Foster City, CA, USA), DNAse/RNAse free water (Sigma, The Netherlands), 400 nM SARS-CoV-2 forward and reverse primer, 200 nM SARS-CoV-2 probe, 300 nM PDV forward primer (5’-cgggtgccttttacaagaac), 300 nM PDV reverse primer (5’-ttctttcctcaacctcgtcc) and100nM PDV probe (NED-aag ggc caa ttc t-MGBNFQ). The ABI PRISM 7500 (Life technologies, USA) was used for the amplification and detection using the profile of 2 min 50 °C, 20 s 95 °C, followed by 45 cycles of 3 s 95 °C and 32 s 60 °C. Since the RNA from kidney and lung tissues were isolated without the addition of PDV as described above, the PDV reaction was in this case redundant.

### Gene expression analysis by RT-qPCR

RNA was reverse transcribed using SuperScript® III reverse transcriptase (Invitrogen, Breda, The Netherlands) and random hexamer primers (Promega, Leiden, The Netherlands). Next, complementary DNA (10 ng) was used for quantitative polymerase chain reaction (RT-qPCR) using a ViiA7 real-time PCR System (Applied Biosystems, Nieuwerkerk aan den IJssel, The Netherlands). Details of the assay-on-demand primers (ThermoFisher Scientific) used in this study are described in Additional file 1: Table S2. Duplicate real-time PCR analyses were performed for each sample, and the obtained threshold cycle (CT) values were averaged. Gene expression values were normalized to the expression of the housekeeping gene GAPDH, resulting in the ∆CT value. GAPDH is an invariant endogenous control in the assay that corrects for sample-to-sample variations in RT-PCR efficiency. The relative mRNA level was calculated by 2^−∆CT^.

### Statistical analysis

Statistical analyses were performed using GraphPad Prism Software v8. Data are presented as mean ± SD. One-way analysis of variance (ANOVA) followed by Tukey’s multiple comparison test was used to compare the data from bacterial sepsis and COVID-19 patients and controls. Differences were considered significant when *p* < 0.05.

## Results

### Patient characteristics

**Fig. 1:**
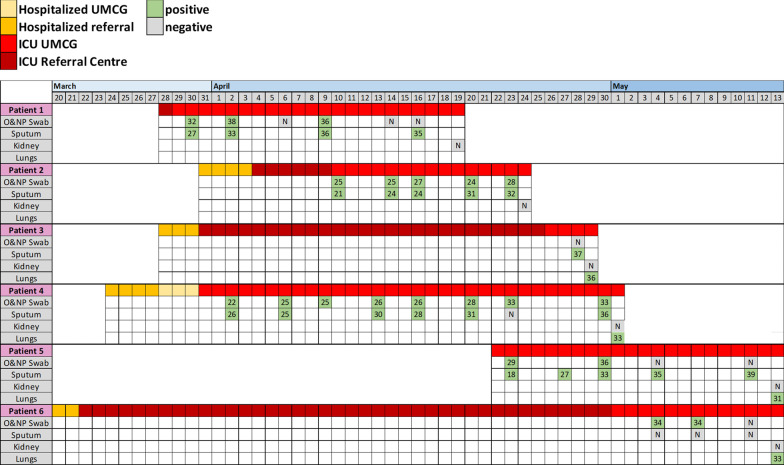
Timeline of hospitalization and SARS-CoV-2 detection. Patients were admitted to the ICU directly or were first hospitalized and went to on to require ICU admission due to the development of severe respiratory failure. Most patients were admitted to the UMCG from a referral hospital. Oropharyngeal and nasopharyngeal swabs and sputum from patients were regularly tested for SARS-CoV-2 virus detection by rRT-PCR during their ICU stay. The presence of SARS-CoV-2 in postmortem lung and kidney biopsies was also determined by rRT-PCR. The cycle threshold (Ct) values are included, N = negative

Postmortem biopsies were collected from patients that died in the ICU with organ failure related to either COVID-19 or bacterial sepsis. Patient characteristics are given in Table 1. Most patients included in our study were male (66% with COVID-19) and (62% with bacterial sepsis). However, the length of ICU stay clearly differed between the groups with the mean length of ICU stay being 30 days for COVID-19 patients (range 21–53) and 3–4 days (range 1–12) for patients with bacterial sepsis. Most, but not all, patients with bacterial sepsis were overweight (66%), yet all patients with COVID-19 included in our study were either overweight or obese. All patients with sepsis had at least one comorbidity, whereas 2 COVID-19 patients had type 2 diabetes mellitus with one of them also having hypertension. All patients, COVID-19 and sepsis were mechanically ventilated. Four out of 6 patients were dialysis-dependent at the time of death. The other 2 patients had a creatinine level of 29 and 68 µmol/L, respectively. One of the patients had a maximum creatinine of 175 µmol/L during ICU stay but was reduced to 68 µmol/L at the time of death. Most patients were admitted to the University Medical Center Groningen (UMCG) from a referral hospital. Oropharyngeal and nasopharyngeal swabs and sputum from patients were regularly tested for SARS-CoV-2 virus detection by rRT-PCR during their ICU stay. The presence of SARS-CoV-2 in postmortem lung and kidney biopsies was also determined by rRT-PCR (Fig. 1). We did not find SARS-CoV-2 RNA in the kidneys of COVID-19 patients in our cohort but did detect SARS-CoV-2 RNA in oro- and naso-pharyngeal swabs, sputum and in lung biopsies from the same patients (Fig. 1). The presence of bacterial co-infection was checked twice a week on a routine basis in all our patients. The patients routinely received a third-generation cephalosporin during the first 4 days of admission and continuous treatment of SDD paste (topical application of tobramycin, colistin, and amphotericin B in the oropharynx and stomach), as part of the selective decontamination of the digestive tract (SDD) treatment [17]. Three patients had a CVL or ECLS cannula-related infection with a coagulase-negative staphylococcus which were treated with vancomycin and line replacement.

### COVID-19-associated AKI is associated with severe and distinct histopathology

**Fig. 2:**
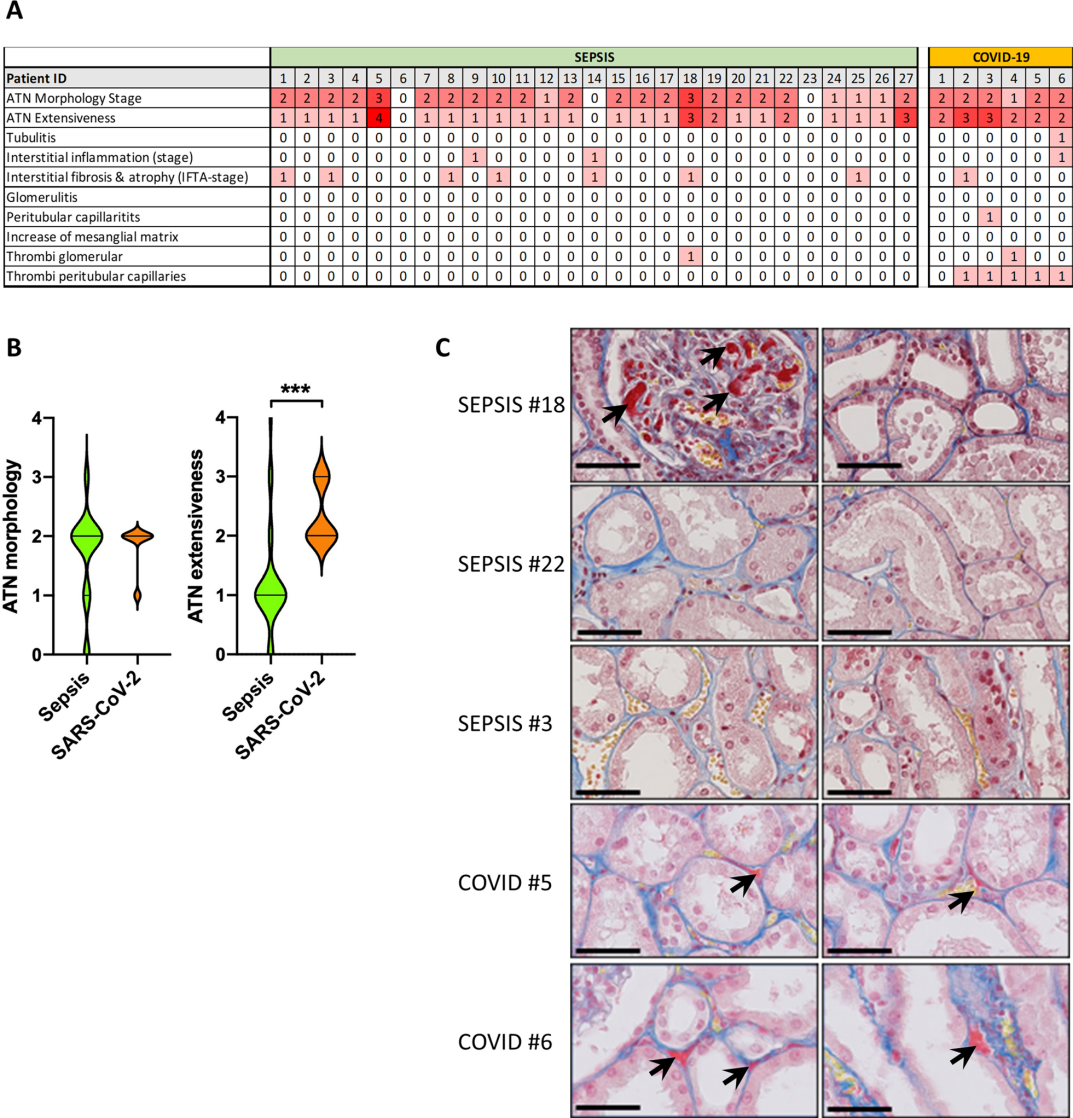
**a** Renal histopathology findings in postmortem biopsies from bacterial sepsis and COVID-19 patients. Histopathology was scored using the scoring system described in Additional file 1: Table S1. **b** Quantification of acute tubular necrosis (ATN) morphology and ATN extensiveness in bacterial sepsis (*n* = 27) and COVID-19 patients (*n* = 6). **c** Representative Martius Scarlett Blue histochemical staining of kidney biopsies from bacterial sepsis and COVID-19 patients. Representative images were taken from randomly selected patients. Arrows indicate microthrombi. Original magnification × 400

We compared the renal histopathology from COVID-19 patients with bacterial sepsis-associated AKI patients (Fig. 2a). We found no evidence of glomerulopathy; tubulitis was observed in 1/6 COVID-19 patients and interstitial inflammation observed in 2/27 patients with bacterial sepsis and 1/6 with COVID-19. Interstitial fibrosis and atrophy were observed in 7/27 patients with bacterial sepsis and 1/6 with COVID-19. One of the most striking observations was the presence of thrombi in 5/6 COVID-19 patients in the renal peritubular capillaries which was absent in biopsies from bacterial sepsis-AKI patients (Fig. 2a, c). Thrombi were additionally found in the glomeruli of 1 COVID-19 patient and 1 patient with sepsis. (Fig. 2a, c). An additional major finding was the extent of acute tubular necrosis (ATN). A degree of ATN was found in most, but not all, patients with bacterial sepsis, yet was present in all biopsies from COVID-19 patients. Morphology stage 2 ATN was the most common in both bacterial sepsis and COVID-19 patients and is associated with tubular vacuolization, tubular edema, epithelial cell flattening and some tubular cell apoptosis. ATN was found in small discontinuous groups of tubules throughout the renal tissue of 24/27 bacterial sepsis patients. In contrast, all renal biopsies from COVID-19 patients had ATN which was also more extensively distributed throughout the total tissue area. Interestingly, we found extensive ATN in renal biopsies from 1 COVID-19 patient that did not have evidence of peritubular capillary thrombosis and was not clinically diagnosed with AKI implying that loss of renal tissue integrity precedes loss of renal function and besides ischemia, multiple other mechanisms might drive ATN (Fig. 2b). Importantly, we did not observe autolysis in our biopsies which allowed for an accurate quantification of ATN severity.

### Renal ACE2 mRNA downregulation is not specific to COVID-19

**Fig. 3:**
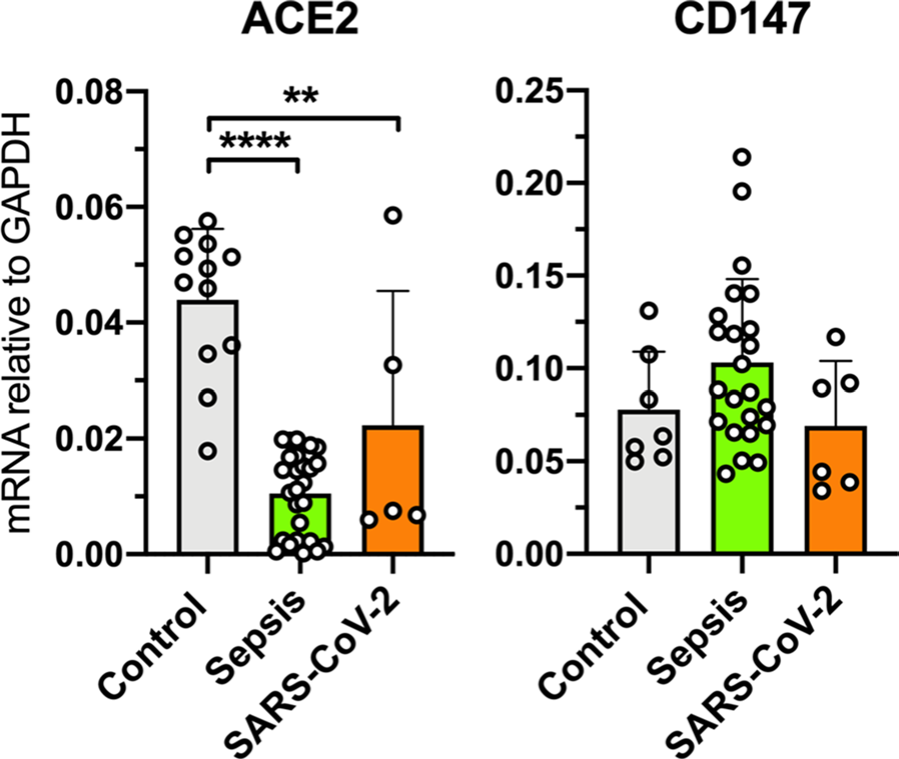
Renal tissue ACE2 and CD147 mRNA levels in critically ill patients with COVID-19-associated AKI (SARS-CoV-2) (*n* = 6), bacterial sepsis-associated acute kidney injury (Sepsis) and control subjects (*n* = 12). Messenger RNA (mRNA) expression was determined by reverse transcription quantitative polymerase chain reaction using glyceraldehyde-3-phosphate dehydrogenase (GAPDH) as a housekeeping gene. **p < 0.01, ****p < 0.0001

ACE2 receptors are expressed in various cell types present in the kidney and are thought to be the main functional receptor for SARS-CoV-2 [18]. However, other receptors such as CD147 have also been implicated to play a role in some cell types [19]. We found low renal ACE2 mRNA levels in both COVID-19 (fold change 0.42, *p* = 0.0002) and bacterial sepsis patients (fold change 0.24, *p* < 0.0001) compared to control, whereas the mRNA levels of CD147 remained unaltered in renal tissue from COVID-19 and bacterial sepsis patients compared to control (Fig. 3). These findings suggest that renal ACE2 mRNA downregulation is not specific to COVID-19 and might be a common feature of AKI in critically ill patients.

### Renal injury and inflammatory biomarker mRNA levels differ in critically ill patients with COVID-AKI from those associated with bacterial Sepsis-AKI

**Fig. 4:**
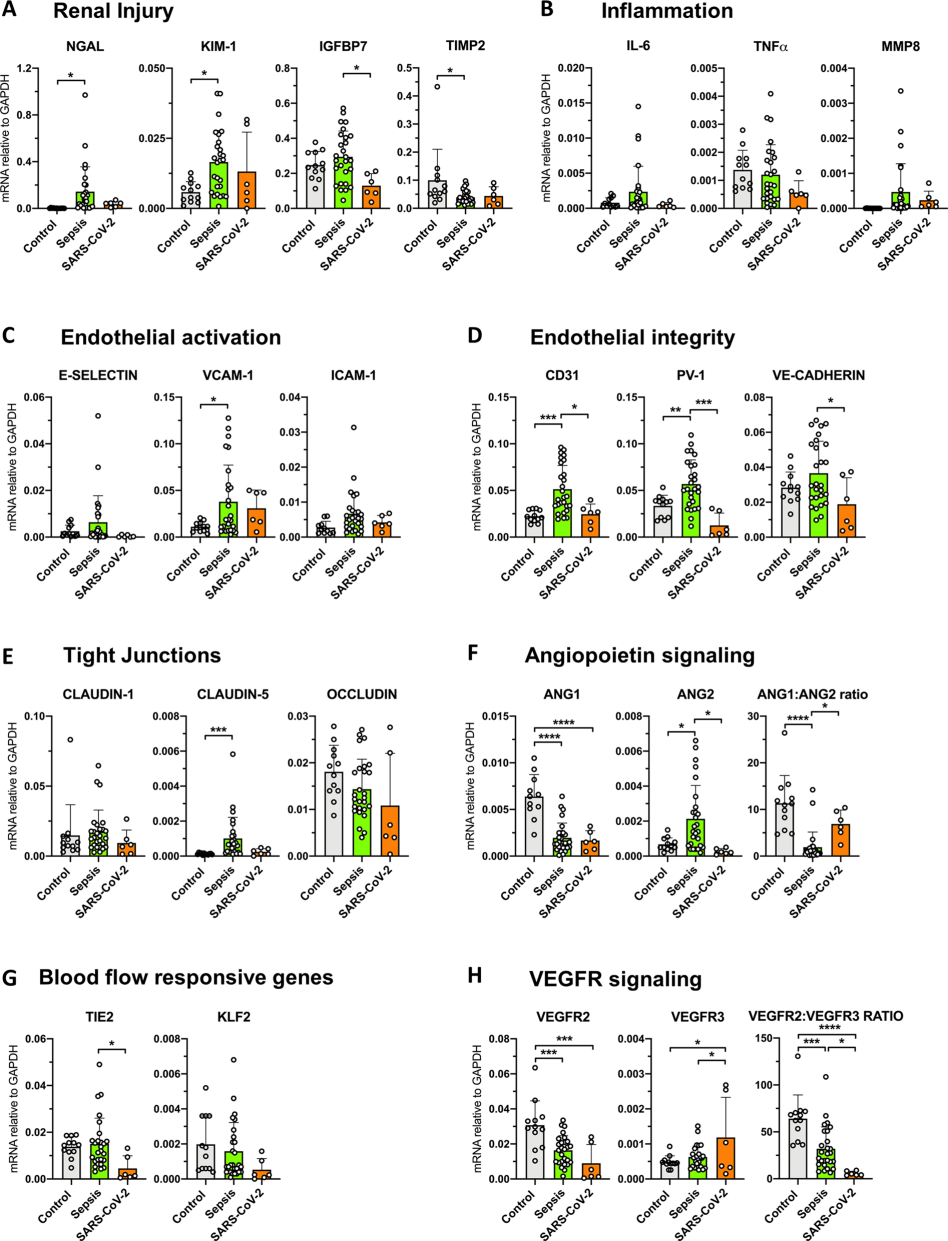
**a**–**h** Renal mRNA levels of various genes in critically ill patients with COVID-19-associated AKI (SARS-CoV-2) (*n* = 6), bacterial sepsis-associated acute kidney injury (Sepsis) and control subjects (*n* = 12). Messenger RNA (mRNA) expression was determined by reverse transcription quantitative polymerase chain reaction using glyceraldehyde-3-phosphate dehydrogenase (GAPDH) as a housekeeping gene. *p < 0.05, **p < 0.01, ***p < 0.001, ****p < 0.0001

In order to gain more knowledge on the possible mechanisms of renal failure in patients with COVID-19 and how that might result in the observed histopathology, we investigated the renal mRNA levels of genes involved in various cellular processes associated with renal failure. Since the extent of tubular injury in COVID-19 is more severe compared to patients with bacterial sepsis, we assumed that the mRNA levels of renal injury markers would be altered. However, renal NGAL and KIM-1 mRNA levels in COVID-19 patients were comparable to normal renal tissue levels yet were increased in patients with bacterial sepsis (NGAL fold change 35, *p* = 0.046, and KIM-1 fold change 24, *p* = 0.012) (Fig. 4a). IGFBP7 mRNA levels were unaltered in patients with bacterial sepsis but were low in renal tissue from COVID-19 patients (fold change 0.46, *p* = 0.014). In contrast, renal TIMP-2 mRNA levels were low in bacterial sepsis (fold change 0.4, *p* = 0.017) but not COVID-19 patients compared to normal controls (Fig. 4a). NGAL is known to be inflammation-driven, and the lack of NGAL upregulation might be attributed to a lack of renal inflammation since in COVID-19 patients the mRNA levels of renal IL-6, TNFα and MMP8 were similar to controls (Fig. 4b). Therefore, in COVID-19, the expression of renal injury and inflammatory markers suggests a different pattern of renal injury to that found in patients with bacterial-sepsis. However, renal stress and injury responses are dynamic, and these findings could therefore be related to the timing of biopsy after disease onset.

### COVID-19-AKI is associated with distinct endothelial responses

We proceeded to investigate genes regulating endothelial inflammatory activation and barrier integrity. Endothelial activation was absent in renal tissue from COVID-19 patients as indicated by the lack of E-selectin, VCAM-1 and ICAM-1 upregulation (Fig. 4c). Renal mRNA levels of CD31, PV-1 and VE-Cadherin did not differ from control individuals yet were increased in bacterial sepsis patients (CD31 fold change 2.3, *p* = 0.0006, PV-1 fold change 1.5, *p* = 0.008) (Fig. 4d). In the kidney, Claudin-1, Claudin-5 and Occludin have a key role in mediating paracellular permeability to solutes [20, 21]. However, the mRNA levels of these molecules in COVID-19 and bacterial sepsis were comparable to control individuals (Fig. 4e). Ang1 mRNA levels were downregulated in renal tissue from both COVID-19 (fold change 0.27, *p* < 0.0001) and bacterial sepsis patients (fold change 0.67, *p* < 0.0001) compared to controls (Fig. 4f). However, in contrast to patients with bacterial sepsis (fold change 3.1, *p* = 0.0215), renal Ang2 mRNA levels in COVID-19 patients were comparable to controls. The Ang1/Ang2 ratio was therefore not disturbed in COVID-19 patients. Together these data suggest that at the time of death, distinct endothelial gene responses in COVID-19 compared to bacterial sepsis-AKI. However, we examined the final stage of disease and cannot exclude that endothelial responses at an earlier stage during the clinical course of COVID-19 were similar to those observed for bacterial sepsis.

### Renal endothelial mRNA responses suggest impaired renal perfusion in COVID-19

Altered perfusion might be a crucial mechanism associated with AKI which could result in acute tubular necrosis. Tie2 and KLF2 are genes shown to be responsive to altered blood flow [22]. Interestingly, we found Tie2 to be downregulated in renal tissue from COVID-19 patients compared to normal control (fold change 0.33, *p* = 0.037) and bacterial sepsis patients suggesting aberrant renal perfusion in COVID-19-AKI patients (Fig. 4g). We also found a decreased VEGFR2/VEGFR3 ratio in bacterial sepsis (fold change 0.5, *p* < 0.0001) which was even more decreased in COVID-19-AKI (fold change 0.09, *p* < 0.0001) (Fig. 4h). Combined with the decreased Tie2 levels this indeed suggests decreased renal blood flow.

## Discussion

In order to develop better treatments for COVID-19 patients and especially with COVID-19-related AKI, we have to understand the mechanisms underlying these threatening sequelae better. In contrast to many recently published studies describing postmortem kidney pathology findings, we aimed to collect biopsies within 1 h after death since postmortem autodigestion of tissue can compromise histopathological findings and destruct RNA needed for molecular biology studies. Indeed, it was often not possible to accurately determine the degree of acute tubular injury due to severe autolysis in many tissue samples in various studies investigating postmortem kidney pathology [23, 24]. Therefore, we collected biopsies with a mean biopsy time of 21 min after death (range 6–40 min), thereby eliminating major autolytic changes in renal structure.

There is much debate as to whether SARS-CoV-2 can directly infect the kidney [25]. Recent reports in which SARS-CoV-2 RNA or protein was found located within the kidney, claim that direct infection of kidney epithelial cells can occur thereby inducing renal injury [26–31]. Similarly, other studies found SARS-CoV-2 RNA or nucleocapsid protein in the urine of COVID-19 patients which might also suggest direct viral infection of the kidney [32, 33]. However, not all studies examining renal pathology in COVID-19 corroborate these findings, and as an increasing number of studies with contradictory findings appear, it seems more plausible that there are subtypes of COVID-19-AKI: (1) direct viral infection, (2) renal failure as a result of secondary host response effects to SARS-CoV-2 infection, i.e., cytokine-mediated effects, damage-associated molecular pattern-mediated injury, complement-mediated damage [34], coagulation-mediated ischemia, or an as yet unrecognized mechanism leading to renal failure. We did not find SARS-CoV-2 RNA in the kidneys of COVID-19 patients in our cohort but did detect SARS-CoV-2 RNA in oral and nasopharyngeal swabs, as well as in lung biopsies from the same patients. We therefore assume that our patients do not have extra-respiratory viral tropism in the kidney and that we are investigating renal failure that resulted from secondary responses to SARS-CoV-2 infection. However, we examined the final stage of the disease and cannot exclude renal viral tropism at an earlier stage during the clinical course. The lack of renal TIMP-2, IGFBP7 and NGAL upregulation is not likely to be directly related to viral infection. It is known that NGAL is inflammatory-driven, and the lack of inflammation observed at the time of death corresponds with the lack of NGAL upregulation. However, we cannot exclude that renal NGAL or other renal injury and inflammatory markers were not altered at an earlier stage during the clinical course of the COVID-19.

ACE2 receptors are expressed in various cell types present in the kidney and are thought to be the main functional receptor for the SARS-CoV-2 virus [18]. However, other receptors such as CD147 have also been implicated to play a role in some cell types such as endothelial cells [19]. We found reduced renal ACE2 mRNA levels in both COVID-19 and bacterial sepsis patients compared to control, implying that reduced ACE2 mRNA expression is not specific to COVID-19, and that renal ACE2 mRNA downregulation might be a general feature of critically ill AKI patients.

The major histopathological findings in renal biopsies from COVID-19 patients in our cohort were ATN, which was present in all biopsies, and thrombi in the renal peritubular capillaries, which were found in biopsies in 5/6 COVID-19 patients. In other postmortem COVID-19 biopsy studies, ATN is also reported although the extent varies [8, 23, 34–39]. Thrombi in peritubular capillaries were rarely observed in other studies. However, one study reported an incidence of fibrin thrombi of 75% [35]. Three other studies reported incidences of microthrombi varying from 4.5 to 14% [23, 28, 38]. Microthrombi in peritubular capillaries were reported without specification of the incidence in one study [39]. Renal inflammation and endothelial activation were absent in COVID-19 patients as indicated by the lack of inflammatory cytokine and endothelial adhesion molecule upregulation. This may also explain the lack of observed interstitial inflammation in the kidney. Although there were no signs of endothelial activation, impaired endothelial integrity was identified. Renal mRNA levels of CD31, PV-1 and VE-Cadherin were reduced in COVID-19 compared to bacterial sepsis patients. Apart from maintaining endothelial integrity, PV-1 is involved in leukocyte transport and the repair of glomerular and peritubular capillary fenestration [40]. Loss of PV-1 mRNA may therefore mediate impaired endothelial barrier function. The angiopoietin signaling system is known to be important in regulating endothelial homeostasis [41]. When disturbed, inflammatory and hyperpermeability responses resulting in pulmonary and renal failure occur in critically ill patients [42]. Despite a reduction in Ang1 mRNA levels in renal tissue, Ang2 mRNA levels were unaltered and the Ang1/Ang2 ratio undisturbed in COVID-19-AKI patients. In patients with bacterial sepsis, the Ang1/Ang2 ratio is disturbed suggesting that the regulation of the angiopoietin system differs in late COVID-19 to bacterial sepsis. These results might even suggest that targeting this system therapeutically during the late stages of disease might have limited value in COVID-19-associated renal failure.

Microvascular endothelial flow responses are tightly controlled. We found Tie2 to be downregulated in renal tissue from COVID-19 patients compared to normal control and bacterial sepsis patients which was previously shown to be flow-dependent [22]. Apart from Tie2, the VEGF receptors VEGFR2 and VEGFR3 have also been shown to mediate flow responses. Therefore, when flow is disturbed, the VEGFR2/VEGFR3 ratio decreases [43]. We found a decreased VEGFR2/VEGFR3 ratio in bacterial sepsis which was even more decreased in COVID-19-AKI. Together, these results combined with the observed histopathology suggest that aberrant renal perfusion, perhaps as a result of the observed peritubular microthrombi, may contribute to the observed tubular injury and renal failure in COVID-19 patients. Supporting our findings, impaired renal blood flow was found in COVID-19 patients with AKI which was not attributed to changes in cardiac output or right ventricular function [44], implying indeed that renal microvascular dysfunction and microthrombi-based occlusion play a predominant role in COVID-19-AKI. In addition, albeit not in the kidney, decreased blood flow in sublingual capillaries in COVID-19 patients was recently described [45, 46]. Currently, no therapies are available for COVID-19-AKI. Our results and those of others suggest that normalizing and/or repairing the renal microvasculature might be a way to alleviate renal failure in critically ill patients with COVID-19.

Until now, the mRNA levels of genes involved in various cellular mechanisms driving AKI have not yet been investigated in COVID-19 patients. However, we acknowledge the following limitations of our study. First the number of COVID-19 patients in this study is 6 which is low. Additionally, patients with bacterial sepsis usually reside in the ICU for around 3–4 days, whereas COVID-19 patients often remain ICU-bound for 2 or more weeks before recovery or death. At this late stage, inflammation and other pathophysiological mechanisms might have resolved which could have had an impact on the results of this study. However, death is a definitive endpoint with probably more similarity between bacterial sepsis and COVID-19 than any other time point during the clinical course of both diseases. Our findings have identified distinct histopathology and gene expression profiles between bacterial sepsis and late COVID-19 which might have important consequences for patient therapy before death. Due to the limited amount of renal tissue collected via percutaneous biopsies, we were limited in which analyses we could perform. Future studies need to be larger, focus on clinical heterogeneity and should investigate the correlation between mRNA and corresponding protein levels, as well as investigating soluble factors present in the plasma and how these factors relate to renal function.

## Conclusion

COVID-19 was associated with severe ATN and microvascular thrombosis coupled with decreased microvascular flow, yet minimal inflammation. Further studies are required to determine whether these observations are a result of true pathophysiological differences or related to the timing of biopsy after disease onset.

## Supplementary Information

**Additional file 1**. Comparison of renal histopathology and gene expression profiles between severe COVID-19 and bacterial sepsis in critically ill patients.

## Data Availability

The datasets used and/or analyzed during the present study are available from the corresponding author on reasonable request.

## Abbreviations

AKI: acute kidney injury
ICU: intensive care unit
KDIGO: kidney disease improving global outcomes criteria
RIFLE: risk, injury, failure, loss of kidney function, and end-stage kidney disease
SARS-CoV-2: severe acute respiratory syndrome coronavirus 2
