# Supplementary material for: Comparison of renal histopathology and gene expression profiles between severe COVID-19 and bacterial sepsis in critically ill patients
Source: Crit Care. 2021 Jun 10;25:202. doi: 10.1186/s13054-021-03631-4 (PMC8190989; doi:10.1186/s13054-021-03631-4)
Supplement: Supplementary file 1 — Additional file 1. Comparison of renal histopathology and gene expression profiles between severe COVID-19 and bacterial sepsis in critically ill patients. [file 13054_2021_3631_MOESM1_ESM.docx]

**ADDITIONAL FILE 1**

**Comparison of renal histopathology and gene expression profiles between severe COVID-19 and bacterial sepsis in critically ill patients**

Meint Volbeda^1^, Daniela Jou-Valencia^1^, Marius C. van den Heuvel^2^, Marjolein Knoester^3^, Peter J. Zwiers^4^, Janesh Pillay^1^, Stefan P. Berger^5^, Peter H.J. van der Voort^1^, Jan G. Zijlstra^1,4^, Matijs van Meurs^1,4^, Jill Moser^1,4*^

1. Department of Critical Care, University of Groningen, University Medical Center Groningen, Groningen, The Netherlands.

2. Department of Pathology and Medical Biology, Pathology section, University of Groningen, University Medical Center Groningen, Groningen, The Netherlands.

3. Department of Clinical Microbiology and Infection Prevention, University of Groningen, University Medical Center Groningen, Groningen, the Netherlands

4. Department of Pathology and Medical Biology, Medical Biology section, Laboratory for Endothelial Biomedicine & Vascular Drug Targeting Research, University of Groningen, University Medical Center Groningen, Groningen, The Netherlands.

5. Department of Internal Medicine, University of Groningen, University Medical Center Groningen, Groningen, The Netherlands.

**Supplementary Table 1.** Histopathological evaluation of kidney biopsies and scoring methods

**Supplementary Table 2.** Human and mouse Assays-on-Demand primers

**Supplemental Table 1.**

**HISTOPATHOLOGICAL EVALUATION OF KIDNEY BIOPSIES AND SCORING METHODS**

| GLOMERULUS |  |
| --- | --- |
| Glomerular sclerosis: | Positive when total sclerosis of the glomerulus was observed. |
| Glomerulitis: | No: < 10 leukocytes in glomerular capillaries  Yes: > 10 leukocytes in glomerular capillaries |
| Thrombi glomerular: | 0: No, 1: Yes |
| Increase of mesangial matrix: | 0: no mesangial matrix increase  1: up to 25% of non-sclerotic glomeruli affected  2: 26-50% of non-sclerotic glomeruli affected  3: >50% of non-sclerotic glomeruli affected.  All at least moderate matrix increase. |

| TUBULES |  |
| --- | --- |
| Acute Tubular Necrosis (ATN) Morphology: | 0: Absent  1: tubulointerstitial edema, dilatation, thinning tubular epithelia  2: apoptotic tubular epithelial cell, casts, signs of regeneration  3: signs of extensive tubular loss |
| ATN Extensiveness | 1: only rare tubules with signs of ATN  2: small discontinuous groups of tubules with signs of ATN  3: easily found larger groups of tubules with signs of ATN  4: extensive areas of tubules with signs of ATN |
| Tubulitis | 0: no mononuclear cells in tubules  1: 1-4 mononuclear cells/tubular cross-section  2: 5-10 mononuclear cells/tubular cross-section  3: >10 mononuclear cells/tubular cross-section |
| Interstitial Inflammation | 0: no or hardly any mononuclear cells in the interstitial parenchyma  1: 10-25% interstitial parenchyma covered with mononuclear cells  2: 26-50% interstitial parenchyma covered with mononuclear cells  3: >50% interstitial parenchyma covered with mononuclear cells. |
| Interstitial fibrosis & tubular atrophy (IFTA) | 0: absent  1: (mild), <25%  2: (moderate), 25-50%  3: (severe), >50% of the total area. |

| MICROVASCULATURE |  |
| --- | --- |
| Intima sclerosis  (% vessel lumen narrowing) | 0: none,  1: <25%  2: 25-50%  3: >50% |
| Intima arteritis | 0: none  1: <25% vessel narrowing  2: >25% vessel narrowing  3: transmural arteritis, fibrinoid changes and media necrosis |
| Arteriolar hyaline | 0: none  1: small-moderate  2: moderate-severe in multiple arteries  3: severe in most arteries |
| Peritubular capillaritis | 0: <10% capillaries containing inflammatory cells  1: >10% capillaries containing 3-4 luminal cells  2: >10% capillaries containing 5-10 luminal cells  3: >10% capillaries containing >10 luminal cells. |
| Thrombi peritubular capillaries | 0: No  1: Yes |

**Supplemental Table 2.**

The following Assay-on-Demand primers were used in this study (ThermoFisher Scientific).

| **Name** | **Assay ID** |
| --- | --- |
| **GAPDH** | Hs99999905_m1 |
| **ACE2** | Hs01085333_m1 |
| **CD147** | Hs01085333_m1 |
| **NGAL** | Hs01008571_m1 |
| **KIM-1** | Hs03054855_g1 |
| **IGFBP7** | Hs00266026_m1 |
| **TIMP-2** | Hs00234278_m1 |
| **IL-6** | Hs00174131_m1 |
| **IL-8** | Hs00174103_m1 |
| **TNFα** | Hs00174128_m1 |
| **MMP-8** | Hs01029052_m1 |
| **E-selectin** | Hs00174057_m1 |
| **VCAM-1** | Hs00365486_m1 |
| **ICAM-1** | Hs00164932_m1 |
| **CD31** | Hs00169777_m1 |
| **PV-1** | Hs00229941_m1 |
| **VE-cadherin** | Hs00174344_m1 |
| **Claudin-1** | Hs00221623_m1 |
| **Claudin-5** | Hs00533949_s1 |
| **Occludin** | Hs00170162_m1 |
| **Ang1** | Hs00181613_m1 |
| **Ang2** | Hs00169867_m1 |
| **Tie2** | Hs00176096_m1 |
| **KLF2** | Hs00360439_g1 |
| **VEGFR2** | Hs00176676_m1 |
| **VEGFR3** | Hs01047677_m1 |
